# Aberrant synaptophysin expression in classic Hodgkin lymphoma

**DOI:** 10.1186/s13000-022-01272-x

**Published:** 2022-11-18

**Authors:** Soyoung Im, Jeong-A. Kim, Gyeongsin Park, Uiju Cho

**Affiliations:** 1 Department of Pathology, St. Vincent’s Hospital, College of Medicine, The Catholic University of Korea, 222 Banpo-daero, Seocho-gu, Seoul, Republic of Korea; 2 Department of Internal Medicine, St. Vincent’s Hospital, College of Medicine, The Catholic University of Korea, Seoul, Republic of Korea; 3 Department of Pathology, Seoul St. Mary’s Hospital, College of Medicine, The Catholic University of Korea, Seoul, Republic of Korea

**Keywords:** Synaptophysin, Hodgkin lymphoma, Diagnosis

## Abstract

**Background:**

Synaptophysin is an immunohistochemical marker for neuroendocrine differentiation and is widely used in pathologic diagnosis. Its expression in malignant lymphoma has not yet been described. However, we experienced an index case of classic Hodgkin lymphoma with synaptophysin expression. This experience prompted us to investigate synaptophysin expression in classic Hodgkin lymphoma.

**Method:**

Immunohistochemical staining of synaptophysin was performed in 59 diagnosed cases of classic Hodgkin lymphoma, 10 anaplastic large cell lymphomas, 16 diffuse large B-cell lymphomas, and 5 extranodal marginal zone lymphoma of the mucosa-associated tissue. Synaptophysin-positive cases were stained for both chromogranin and CD56a.

**Result:**

Of 59 classic Hodgkin lymphoma cases, 11 (19%) were positive for synaptophysin. None of the anaplastic large cell lymphomas expressed synaptophysin. Synaptophysin showed weak but specific expression in the cytoplasm of the Hodgkin lymphoma tumor cells. Other background inflammatory cells (such as macrophages, B-, and T-lymphocytes) were all negative for synaptophysin expression. Chromogranin and CD56a were not expressed in the synaptophysin-positive classic Hodgkin lymphomas.

**Conclusions:**

Synaptophysin is an integral glycoprotein present in presynaptic vesicles of neurons and neuroendocrine cells. It is a diagnostic marker for neuroendocrine tumors. Aberrant synaptophysin expression has been reported in non-neuroendocrine tumors but not in lymphoma or leukemia. To the best of our knowledge, synaptophysin positivity has only been reported in a single case of precursor T-lymphoblastic leukemia/lymphoma to date. Our study showed that aberrant synaptophysin expression in classic Hodgkin lymphoma is an unexpectedly frequent finding. The mechanism underlying, and prognostic significance of, such aberrant expression is unclear. Thus, in a small biopsy, aberrant synaptophysin expression could be a diagnostic pitfall and should be carefully avoided.

## Background

Synaptophysin is an integral membrane glycoprotein in presynaptic vesicles. This protein is present in almost all neurons and is a component of small neuron vesicles. An anti-synaptophysin antibody is expressed in normal neuroendocrine cells and various tumors, such as adrenocortical tumors, neuroendocrine carcinomas, Merkel cell carcinoma, paraganglioma, and neuroblastomas, and is used in the diagnosis of tumors. It is expressed in the neurons and neuroendocrine cells of the adrenal medulla, carotid artery, skin, pituitary gland, thyroid, lung, pancreas, and gastrointestinal mucosa, as well as in the benign and malignant tumors occurring in these cells [1–3].

During the diagnosis of a case of Hodgkin lymphoma, we discovered that tumor cells stained positive for anti-synaptophysin antibodies. This index case led us to evaluate in synaptophysin expression in Hodgkin lymphoma. Synaptophysin expression in Hodgkin lymphoma is a novel finding that has not been reported in the literature. To date, synaptophysin expression has not been reported in normal immune cells, and its overexpression in hematologic malignancies, including lymphoma, has not been reported, except in one case of chronic lymphoblastic leukemia/small lymphocytic lymphoma [4]. Synaptophysin and other neuroendocrine tumor markers are also expressed in tumors unrelated to neuroendocrine cells. In particular, the expression of these markers has been found in alveolar rhabdomyosarcoma [5], angiosarcoma [6], and malignant melanoma [7], and it is possible to reduce the risk of diagnosing these cancers as neuroendocrine carcinoma by based on this finding. Further studies of these tumors have identified the overexpression of intermediate filaments unrelated to tumor cells [8, 9]. During the diagnostic process, ancillary immunohistochemical staining is widely used, and pathologists encounter unexpected “aberrant” or “anomalous” protein expression profiles in many tumors. Unexpected protein expression or the loss or expression in malignant tumors may contribute to tumor processes, such as tumorigenesis or immune evasion [10, 11]. It can also be used as a pathological diagnostic marker or be a risk factor for misdiagnosis [12].

Positivity for synaptophysin in Hodgkin lymphoma tumor cells has not been studied. Therefore, this study aims to determine the usefulness of synaptophysin as a diagnostic marker by investigating the frequency of synaptophysin expression in Hodgkin lymphoma.

## Methods

### Case selection

We screened the institutional pathology database of St. Vincent’s Hospital, Suwon, Korea, from January 2010 to December 2021 and identified patients with classic Hodgkin lymphoma and anaplastic large cell lymphoma. Patients for whom formalin-fixed, paraffin-embedded tissue blocks with adequate tissue were available for immunohistochemical analyses were included. The diagnostic slides were reviewed by expert pathologists (U.C. and S.I.). The diagnoses were made according to the revised 4^th^ edition World Health Organization Classification of Tumours of Haematopoietic and Lymphoid Tissues [13]. A tissue microarray slide containing 16 diffuse large B-cell lymphoma and five extranodal marginal zone lymphoma of the mucosa-associated tissue (MALT lymphoma) cases was purchased from US Biomax (HL801a; Rockville, MD, USA). The study was approved by the St. Vincent’s Institutional Review Board (VC19SISI0268).

### Immunohistochemistry

The immunohistochemical stains were performed on paraffin-embedded, whole-tissue sections following standard methods using the Ventana Benchmark XT platform (Ventana Medical Systems, Tucson, AZ, USA). Heat-induced antigen retrieval was performed using the Ventana Benchmark Cell Conditioning solution (CC1) program. Two different primary antibody clones were used for synaptophysin: 1) clone MRQ40 (rabbit monoclonal, 1:100; Cell Marque, Rocklin, CA, USA and 2) clone SP11 (rabbit monoclonal, prediluted; Roche/Ventana, Oro Valley, AZ, USA). Also, primary antibodies CD56 (clone MRQ42, rabbit monoclonal, 1:200; Cell Marque), and chromogranin (clone LK2H10, mouse monoclonal, 1:200; Cell Marque) were used for the study. We tested all the cases for synaptophysin and then performed CD56 and chromogranin staining for the synaptophysin-positive cases. Two pathologists assessed synaptophysin, CD56, and chromogranin cytoplasmic expression (U.C. and S.I.). Cases with ≥ 5% tumor cell expression were interpreted as positive.

### Statistical analysis

Analysis was performed using R software (version 4.1.2.). Categorical variables are reported as number (%) and continuous variables as median (range). Pearson’s chi-square or Fisher’s exact tests were used to compare categorical variables between groups. A two-sided *p*-value < 0.05 was considered significant.

## Results

We studied 59 classic Hodgkin lymphomas (CHL) and ten anaplastic large cell lymphomas (ALCL). The CHL cohort was composed of 36 mixed cellularity (61%), 15 nodular sclerosing (25%), 6 lymphocyte-depleted (10%), and 2 lymphocyte-rich CHL (3%) cases. For CHL, the median age at diagnosis was 53 years old (range: 4–92 y), and 71% (*n* = 42) were male. For ALCL, the median age at diagnosis was 67.5 years (range: 35–82 y), and 80% were male.

Of the total 59 CHL cases, 11 (19%) showed synaptophysin expression (Table 1). The staining results were the same in both clone MRQ40 and SP11 synaptophysin antibodies.

**Table 1:** Immunohistochemical expression of synaptophysin in 59 lymphoma cases

	No. of cases	No. of positive cases (%)
Classic Hodgkin lymphoma	59	11 (19%)
Mixed cellularity type	36	4 (11%)
Nodular sclerosis type	15	5 (33%)
Lymphocyte-depleted type	6	2 (33%)
Lymphocyte-rich type	2	0
Anaplastic large cell lymphoma	10	0
Diffuse large B-cell lymphoma	16	0
MALT lymphoma	5	0

MALT lymphoma, extranodal marginal zone lymphoma of the mucosa-associated tissue

**Fig. 1:**
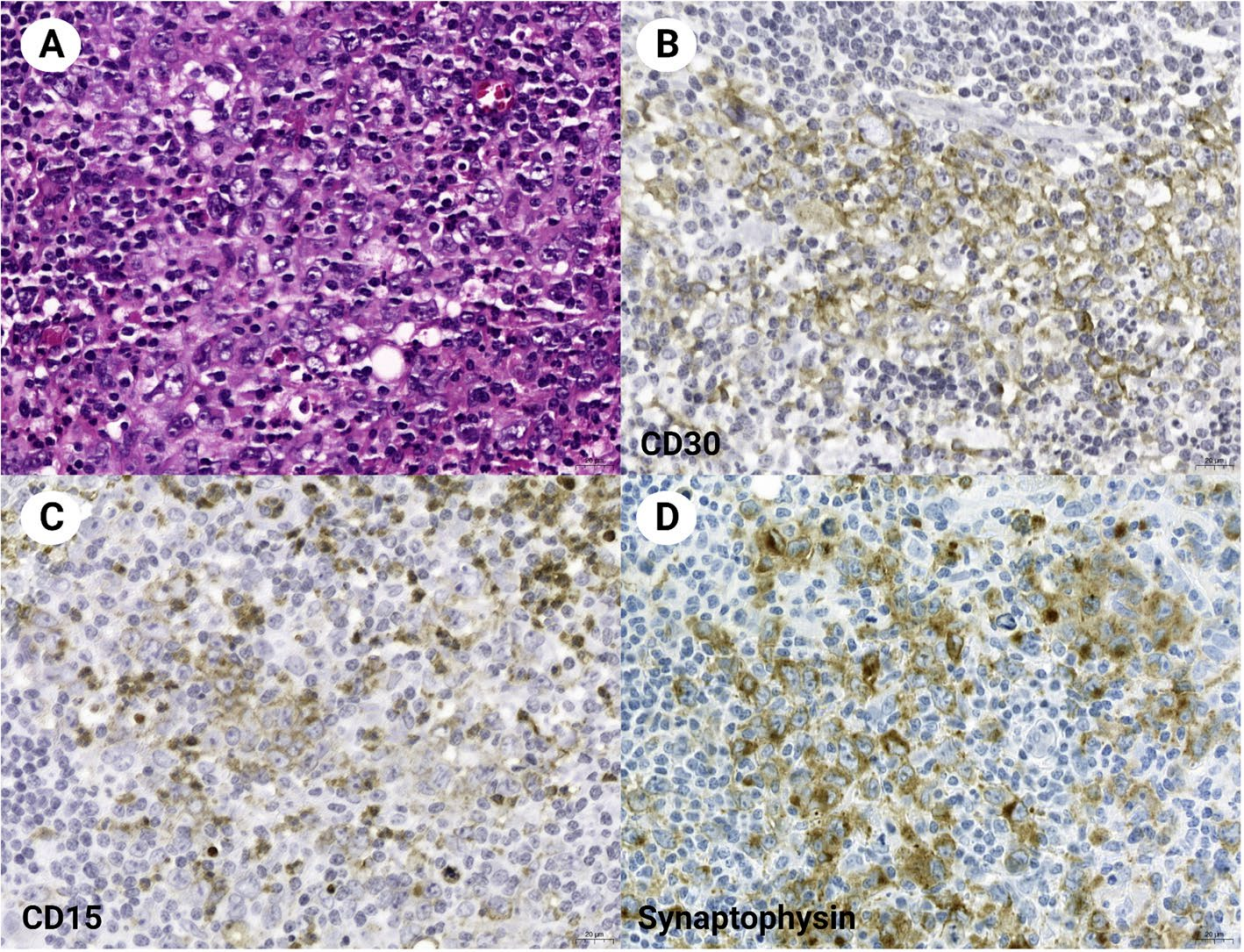
Classic Hodgkin lymphoma involving a mediastinum (Case 1). Light microscopy (A, HE stain) shows Hodgkin/Reed-Sternberg cells with positive staining for CD30 (B), CD15 (C), and synaptophysin (clone MRQ40) (D). Magnification × 400

**Fig. 2:**
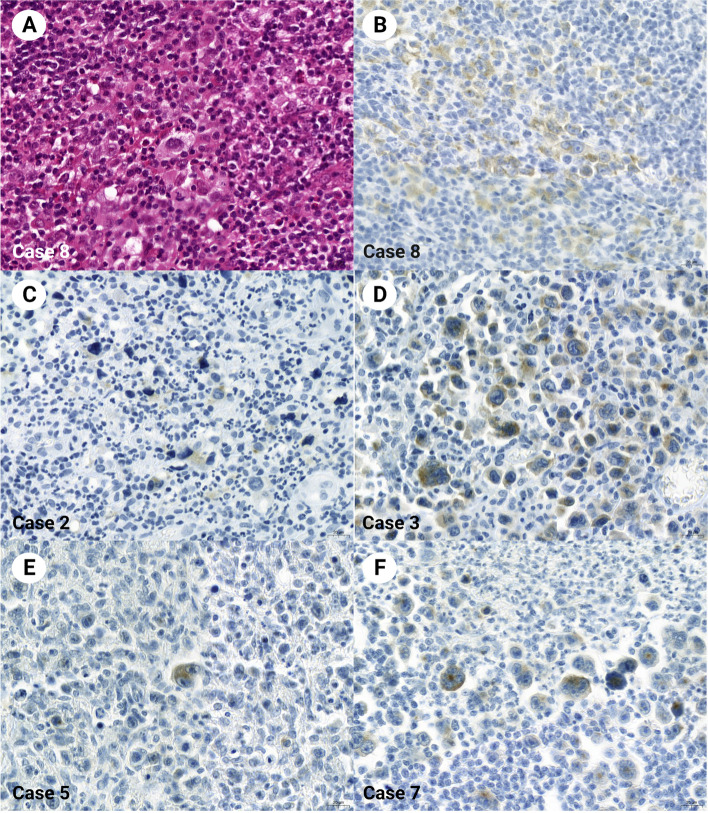
Classic Hodgkin lymphoma with synaptophysin expression. Hodgkin/Reed-Sternberg cells are evident for cytoplasmic synaptophysin expression (A, Case 8, HE stain; B, Case 8, synaptophysin stain). Synaptophysin is stained in the cytoplasm of other Hodgkin/Reed-Sternberg cells of other classic Hodgkin lymphoma cases. The other hematolymphoid cells in the background are all negatively stained (C, Case 2; D, Case 3; E, Case 5; F, Case 7). All were stained with synaptophysin clone MRQ40. Magnification × 400

**Fig. 3:**
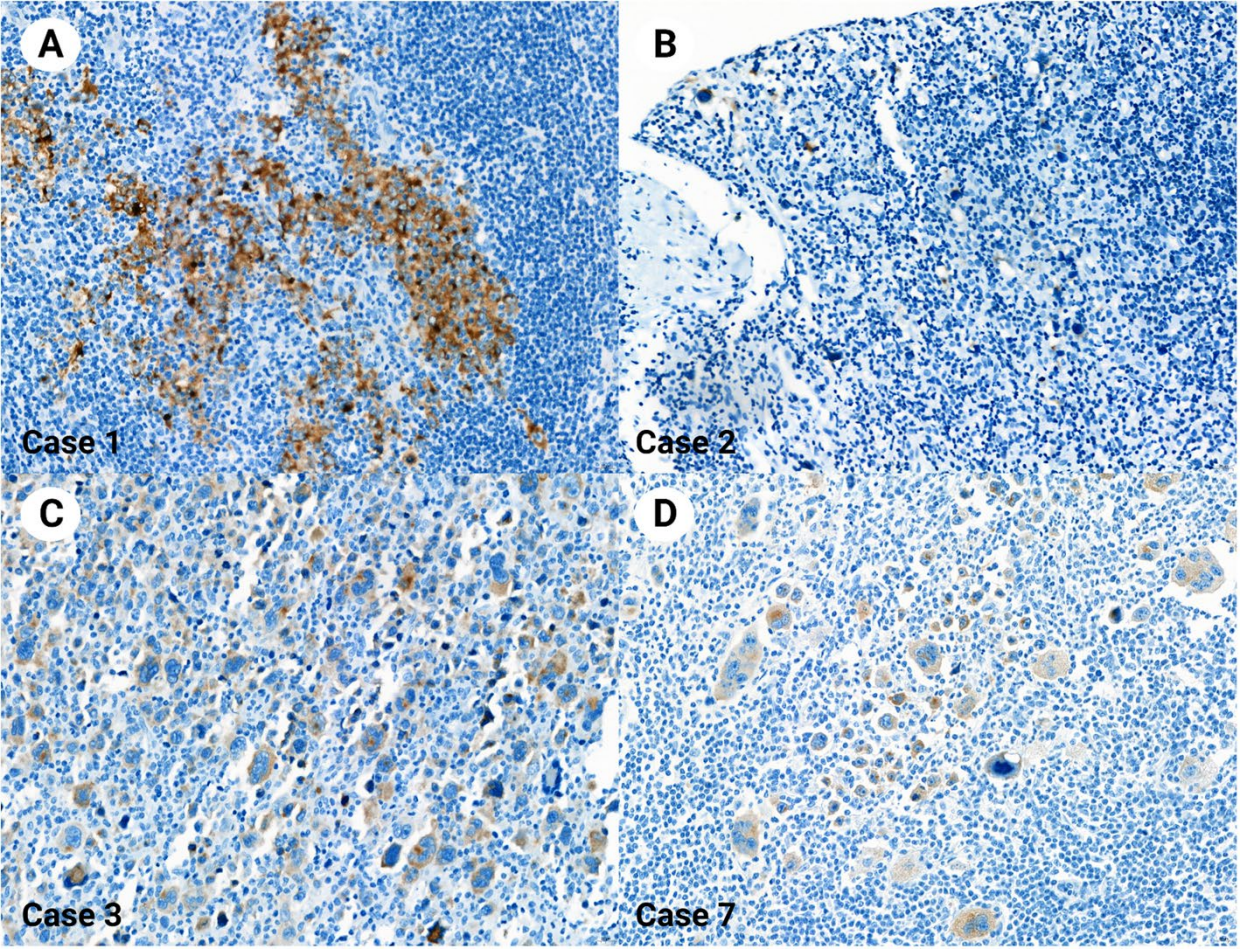
Classic Hodgkin lymphoma stained with synaptophysin clone SP11. The synaptophysin staining contrasts well with the hematoxylin counterstain. The synaptophysin expression pattern was similar to the staining results using clone MRQ40 (A, Case 1; B, Case 2; C, Case 3; D, Case 7). Magnification × 400

In CHL cases, synaptophysin was specifically expressed in Hodgkin Reed-Sternberg (HRS) cells. Synaptophysin expression in HRS cells was evident in the cytoplasm, in weak to moderate intensity, in a fine, granular pattern (Figs. 1 and 2). The expression pattern was the same as in neuroendocrine cells but generally weaker in intensity. No synaptophysin expression was observed in immunoblasts or other B-, T-, and histiocytes in the tumor (Figs. 1 and 2). The number of HRS cells that stained positive for synaptophysin ranged from 50 to 100% (Table 2). The staining of synaptophysin clone SP11 was slightly clearer and brighter than that of clone MRQ40. However, the relative staining intensity and proportion of the positive cells between the cases were virtually identical (Fig. 3).

**Table 2:** Clinicopathologic characteristics of synaptophysin-positive classic Hodgkin lymphoma cases using clone MRQ40

Case no	Age/Sex	Synaptophysin staining intensity	Synaptophysin positive cell proportion	CD56 expression	Chromogranin expression	Tumor site	Histologic type	Specimen type
Case 1	20/F	moderate	100%	Negative	Negative	Cervical lymph node	Nodular sclerosis	Excision
Case 2	36/M	weak	50%	Negative	Negative	Mediastinum	Mixed cellularity	Biopsy
Case 3	21/F	weak	60%	Negative	Negative	Cervical lymph node	Lymphocyte-depleted	Excision
Case 4	28/M	weak	20%	Negative	Negative	Cervical lymph node	Nodular sclerosis	Excision
Case 5	77/F	weak	5%	Negative	Negative	Cervical lymph node	Lymphocyte-depleted	Excision
Case 6	30/M	weak	60%	Negative	Negative	Inguinal lymph node	Mixed cellularity	Excision
Case 7	45/F	weak to moderate	70%	Negative	Negative	Axillary lymph node	Nodular sclerosis	Excision
Case 8	20/M	weak	80%	Negative	Negative	Cervical lymph node	Nodular sclerosis	Excision
Case 9	37/M	weak	70%	Negative	Negative	Mediastinum	Mixed cellularity	Excision
Case 10	54/F	weak	50%	Negative	Negative	Mediastinum	Nodular sclerosis	Biopsy
Case 11	26/F	weak to moderate	70%	Negative	Negative	Cervical lymph node	Mixed cellularity	Biopsy

Both chromogranin and CD56 staining were negative in all synaptophysin-positive CHL cases (Table 2; Fig. 1). There was no association between synaptophysin expression and clinicopathologic characteristics such as age, sex, and histological type. Since many patients did not have progressive observation data, the significance of synaptophysin expression as a prognostic marker could not be analyzed.

No detectable expression was evident in anaplastic large cell, diffuse large B-cell, or MALT lymphomas (Table 1). There was no synaptophysin expression in the background hematopoietic cells with the tumor cells. As a diagnostic marker to differentiate from non-Hodgkin lymphoma, sensitivity was 18.6%, and specificity was 100%. The sensitivity and specificity of synaptophysin as an immunohistochemical marker to distinguish CHL from non-CHL were 18.6% and 100%, with positive and negative predictive values of 100% and 39.2%, respectively (Table 3).

**Table 3:** Predictive values of synaptophysin immunohistochemistry in diagnosing classic Hodgkin lymphomas

Synaptophysin expression	Classic Hodgkin lymphoma	Non- classic Hodgkin lymphoma^*^	Predictive values
Positive	11	0	PPV = 1
Negative	48	31	NPV = 0.392
	Sensitivity = 0.186	Specificity = 1	

^*^Non-classic Hodgkin lymphoma cases included diffuse large B-cell lymphoma, anaplastic large cell lymphoma, and extranodal marginal zone lymphoma of the mucosa-associated tissue

*PPV* positive predictive value, *NPV* negative predictive value

## Discussion

Although synaptophysin is a diagnostic marker used routinely in pathological diagnosis, synaptophysin expression in Hodgkin lymphoma has not been previously described. Of all lymphomas, only one case was discovered accidentally in T-lymphoblastic leukemia/lymphoma [4]. It was an unexpected, novel finding that synaptophysin was not expressed in other reactive hematolymphoid cells in the background, even in cases of CD30-positive anaplastic large cell lymphoma (ALCL), diffuse large B-cell lymphoma (DLBCL) with B-cell lineage, and MALT lymphoma.

Although Hodgkin lymphoma shows a lower incidence than non-Hodgkin lymphoma, it can be cured in more than 75% of cases and more than 50% of patients even if the disease has progressed because its response to chemotherapy is excellent [14, 15]. Accurate diagnosis is particularly important so that appropriate treatment can be started at an early stage. Unlike other lymphomas, Reed-Sternberg (RS) cells (tumor cells of Hodgkin lymphoma) do not occupy most of the lesions but are sporadically distributed between the abundant non-tumor immune cells. Hence, it is crucial to identify the properties of tumor cells with immunohistochemical staining during diagnosis [16]. Diagnosis cannot be based on only one diagnostic marker. In other words, several other markers, such as CD30, CD15, Pax5, MUM1, Fascin, CCl17, CD45, OCT2, and BOB1, are selected and combined for diagnosis. CD30 has the highest expression rate, showing positivity in more than 90% of classic Hodgkin lymphomas [16, 17]. However, CD30 positively stains not only Hodgkin lymphoma cells but also some granulocytes and plasma cells expressed in activated B and T-origin immunoblasts; further, it is overexpressed in natural killer cells and monocytes [18]. It is also strongly expressed in ALCL and is partially expressed in other lymphoma cells. Much attention is required in interpreting the immunostaining results because CD15, Pax5, MUM1, and Fascin are expressed in other immune cells. Markers such as CD45, OCT2, and BOB1, which can be used for diagnosis because they are negative in classic Hodgkin lymphoma, are also positive in many other immune cells in the vicinity of tumor cells [16, 18, 19].

It is not difficult to differentiate classic Hodgkin lymphoma from common forms of neuroendocrine or adrenal cortical tumors. Therefore, aberrant synaptophysin expression would not cause diagnostic confusion, especially when the diagnostic tissue is sufficient. However, it can be a diagnostic pitfall if the pathologist does not consider the possibility of a classic Hodgkin lymphoma in an unusual clinical setting or if synaptophysin staining was performed initially when the amount of diagnostic tissue was very small. These findings are meaningful in that misdiagnosis, or unnecessary additional examinations can be avoided by recognizing that synaptophysin can be positive in HRS cells. Even if the expression rate of synaptophysin is low, it is negative in other immune cells, at least in our study. This characteristic may be used to improve the accuracy of the diagnosis. For example, in ALCL, when it is challenging to diagnose CD30-positive lymphoma or differentiate it from activated immunoblasts, synaptophysin staining may be helpful. Although the sensitivity is low, the specificity for synaptophysin is high. Thus, if synaptophysin is positive along with CD30, it may help render a diagnosis of classic Hodgkin lymphoma. However, further study with a larger number of lymphoma cases, especially those with CD30 expression, is needed to validate that synaptophysin staining appears only in the Hodgkin cells because the number of ALCL cases in our study is only ten, which is very not enough to draw a conclusion confidently.

We performed synaptophysin immunostaining using two common clones, i.e., MRQ40 and SP11, to test whether the difference in synaptophysin clones may produce different staining results. Differences in antibody clones did not result in a significant difference, at least with regard to these two clones. The mechanism underlying the expression of synaptophysin in HRS cells is still unknown. In the present study, other neuroendocrine markers (CD56a and chromogranin) are not expressed, suggesting that HRS cells do not undergo neuroendocrine phenotyping or differentiation. Synaptophysin is generally believed to be expressed in tumors with neural or neuroendocrine differentiation [2]. However, there have been reports of abnormal expression in alveolar rhabdomyosarcoma, extraskeletal myxoid chondrosarcoma, melanoma, and angiosarcoma, and nonspecific or abnormal expression in tumors such as lung adenocarcinoma [5, 6, 12, 20, 21]. The mechanism underlying such an abnormal expression occurs has not yet been revealed. It could be a synaptophysin protein expression, cross-reaction to other antigens, or nonspecific staining. Mechanisms occurring in neoplastic cells, such as mutations and antigen uptake, may result in nonspecific staining patterns different from those of normal cells.

Evidence supports that HRS cells are of the B-cell lineage and arise from germinal or immediate postgerminal centers [22]. Alterations, such as immunoglobulin gene mutation, methylation of B-cell differentiation genes, and the expression of B-cell repressors, render HRS cells highly defective B-cells without functional surface B-cell receptors [22]. Normally, faulty B-cells undergo apoptosis, but HRS cells can evade the apoptotic pathway via incompletely understood mechanisms [22]. The aberrant synaptophysin expression could result from alterations during HRS cell development; however, this process needs to be investigated in future studies.

Interestingly, it has been previously reported that restin, an intermediate filament-associated protein, is overexpressed in RS cells of Hodgkin lymphoma [8]. There have been no further studies on the expression of other intermediate filaments or similar intermediate filament-associated proteins. More extensive studies are needed to ascertain whether proteins such as restin, as well as synaptophysin, are expressed in other hematolymphoid tumors, and follow-up studies on the mechanisms underlying synaptophysin expression will facilitate the diagnosis and understanding of Hodgkin lymphoma.

There are caveats associated with the use of synaptophysin expression for the diagnosis of classic Hodgkin lymphoma. The intensity of synaptophysin expression is generally weaker than that of neuroendocrine tumors, and this is a limitation associated with its use in practice. Further, in a few cases, the percentage of synaptophysin-positive tumor cells was as low as 5%. Therefore, synaptophysin-positivity can be easily overlooked and must be closely observed.

## Conclusions

Herein, we describe a novel finding of synaptophysin expression in Hodgkin lymphoma HRS cells. Awareness of this unique finding may also avoid misdiagnosing neuroendocrine tumors in small biopsy specimens. As the sample size of this study is limited, further large-scale investigations are needed. Follow-up studies on the mechanism underlying the expression of synaptophysin would also facilitate the diagnosis and understanding of Hodgkin lymphoma.

## Data Availability

"The dataset supporting the conclusions of this article is included within the report.

## Abbreviations

ALCL: Anaplastic large cell lymphomas
CHL: Classic Hodgkin lymphomas
DLBCL: Diffuse large B-cell lymphoma
HRS: Hodgkin Reed-Sternberg
MALT: Mucosa-associated lymphoid tissue
